# *tert*-Butyl Hydroperoxide (tBHP)-Induced Lipid Peroxidation and Embryonic Defects Resemble Glucose-6-Phosphate Dehydrogenase (G6PD) Deficiency in *C. elegans*

**DOI:** 10.3390/ijms21228688

**Published:** 2020-11-18

**Authors:** Hung-Chi Yang, Hsiang Yu, Tian-Hsiang Ma, Wen-Ye Tjong, Arnold Stern, Daniel Tsun-Yee Chiu

**Affiliations:** 1 Department of Medical Laboratory Science and Biotechnology, Yuanpei University of Medical Technology, Hsinchu 300102, Taiwan; 2 Department of Medical Biotechnology and Laboratory Sciences, College of Medicine, Chang Gung University, Taoyuan 333323, Taiwan; cohsiang@hotmail.com; 3 Graduate Institute of Health Industry Technology, College of Human Ecology, Chang Gung University of Science and Technology, Taoyuan 333324, Taiwan; aeroufo@gmail.com (T.-H.M.); claratania_louisregina@yahoo.com (W.-Y.T.); 4 Grossman School of Medicine, New York University, New York, NY 10016, USA; Arnold.Stern@nyulangone.org; 5 Research Center for Chinese Herbal Medicine, Graduate Institute of Health Industry Technology, College of Human Ecology, Chang Gung University of Science and Technology, Taoyuan 333324, Taiwan; 6 Department of Pediatric Hematology/Oncology, Linkou Chang Gung Memorial Hospital, Taoyuan 333423, Taiwan

**Keywords:** GSPD-1, tBHP, lipid peroxidation, iPLA, germ cell apoptosis, embryogenesis, *C. elegans*

## Abstract

G6PD is required for embryonic development in animals, as severe G6PD deficiency is lethal to mice, zebrafish and nematode. Lipid peroxidation is linked to membrane-associated embryonic defects in *Caenorhabditis elegans* (*C. elegans*). However, the direct link between lipid peroxidation and embryonic lethality has not been established. The aim of this study was to delineate the role of lipid peroxidation in *gspd-1*-knockdown (ortholog of *g6pd*) *C. elegans* during reproduction. *tert*-butyl hydroperoxide (tBHP) was used as an exogenous inducer. Short-term tBHP administration reduced brood size and enhanced germ cell death in *C. elegans*. The altered phenotypes caused by tBHP resembled GSPD-1 deficiency in *C. elegans*. Mechanistically, tBHP-induced malondialdehyde (MDA) production and stimulated calcium-independent phospholipase A_2_ (iPLA) activity, leading to disturbed oogenesis and embryogenesis. The current study provides strong evidence to support the notion that enhanced lipid peroxidation in G6PD deficiency promotes death of germ cells and impairs embryogenesis in *C. elegans*.

## 1. Introduction

Biochemically, the production of nicotinamide adenine dinucleotide phosphate (NADPH) by glucose-6-phosphate dehydrogenase (G6PD) is crucial for cellular reductive biosynthesis and redox homeostasis [1]. NADPH also plays a cytoregulatory role in free radical production through NADPH oxidase and nitric oxide synthase [2]. Clinically, G6PD deficiency in humans is an X-linked disorder affecting 400 million people globally and is highly prevalent across malarial endemic regions [3]. Reduced G6PD activity due to a mutation predisposes G6PD-deficient individuals to red cell disorders, including favism, neonatal jaundice and drug- or infection-induced hemolysis. Besides hemolytic anemia related clinical manifestations, other clinical presentations due to G6PD deficiency have not been thoroughly investigated.

Recently, a preponderance of evidence indicates that G6PD is required for embryonic development in animals, as severe G6PD deficiency is lethal to mice [4]. Similar to the G6PD-deficient murine models, the *gspd-1*-knockdown (ortholog of *g6pd*) nematode *Caenorhabditis elegans* (*C. elegans*) exhibits a severe hatching defect [5]. It also displays multiple embryonic impairments, including abnormal eggshell structure, enhanced permeability, defective polarity and cytokinesis [6]. These membrane-associated defects have been linked to disturbed membrane lipid composition caused by the activation of calcium-independent phospholipase A_2_ (iPLA) due to elevated lipid peroxidation [6].

Lipid peroxidation plays a role in altered lipid metabolism, membrane structural integrity, redox imbalance and survival in *C. elegans* [7,8,9]. The aim of this study was to delineate the role of lipid peroxidation in *gspd-1*-knockdown *C. elegans* during reproduction. To induce lipid peroxidation in *C. elegans*, *tert*-butyl hydroperoxide (tBHP) was used as an exogenous inducer. tBHP, a short-chain organic hydroperoxide, has been commonly used as a lipid hydroperoxide analog to evaluate the mechanism of biological alterations resulting from oxidative damage [10,11].

The current study revealed for the first time that a short-term tBHP administration can reduce brood size similar to that observed with GSPD-1 deficiency in *C. elegans* [5]. Such administration also phenocopies elevated germ cell apoptosis induced by GSPD-1 deficiency. Both malondialdehyde (MDA) and iPLA activity were shown to be increased by tBHP administration, which resembles GSPD-1 deficiency in *C. elegans* [6]. These findings clearly demonstrate that tBHP-mediated lipid peroxidation can render reproductive activities of GSPD-1 deficient *C. elegans* abnormal due to the activation of iPLA by MDA leading to eventual germ cell death and embryonic lethality.

## 2. Results

### 2.1. Temporal Expression of gspd-1 in C. elegans

**Figure 1:**
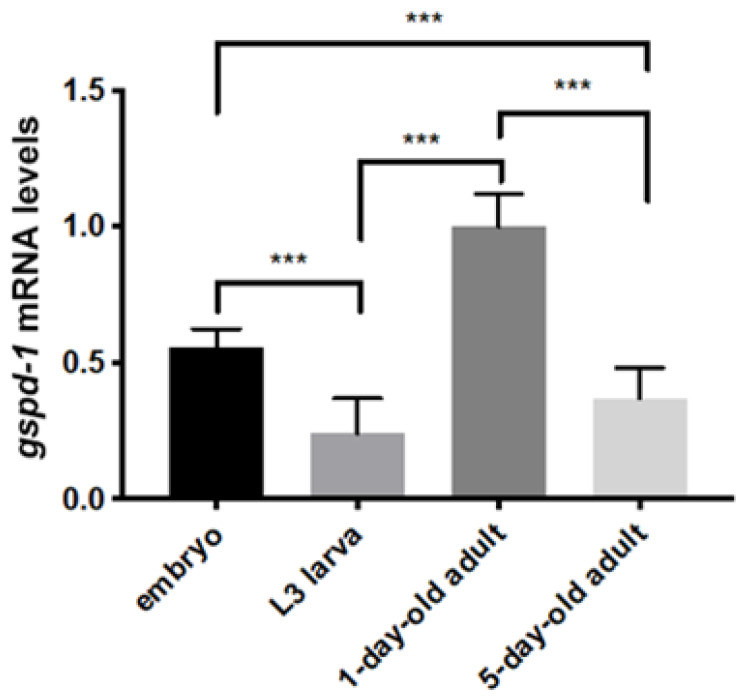
Gene expression pattern of *gspd-1* during development by qPCR. The mRNA expression level of *gspd-1* in the four *C. elegans* stages was analyzed by quantitative PCR. The results are presented as the relative expression level and normalized by *act-1*. Fold change data from three independent experiments are shown as the mean ± SD (*n* = 3, *** *p* < 0.001).

To ensure the quantitative expression of housekeeping gene *gspd-1*, mRNA level of *gspd-1* was measured by qPCR in different stages of *C. elegans* (embryo, L3, one-day-old and five-day-old adults). One-day-old adults contained a mature reproductive system and displayed a high level of oogenesis, while five-day-old adults were relatively old so that egg production was almost ceased and displayed a low level of oogenesis. The temporal expression patterns of *gspd-1* were as follows: *gspd-1* was highly expressed in the one-day-old adult stage (represented as 1.0), followed by moderate expression at the embryonic stage (0.56). Both the L3 stage (0.24) and the five-day-old adult stage (0.37) showed basal levels of *gspd-1* expression (Figure 1). These results suggest that *gspd-1* is ubiquitously expressed in *C. elegans*. The elevated levels of *gspd-1* in the embryonic and young adult stages are consistent with the notion that the function of GSPD-1 is required for embryogenesis and oogenesis.

### 2.2. tBHP Reduced Brood Size in C. elegans

**Figure 2:**
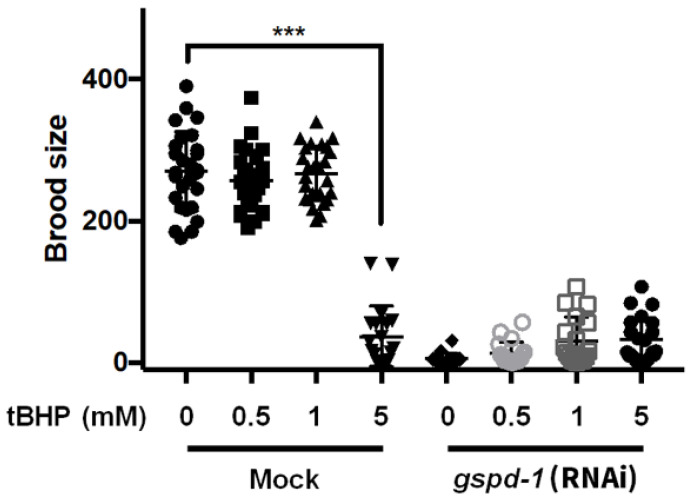
Effect of short-term tBHP administration on brood size. Brood size of mock and *gspd-1*-knockdown *C. elegans* with or without tBHP administration (0.5, 1 and 5 mM) (*** *p* < 0.001).

To study whether lipid peroxidation affected embryonic survival, *C. elegans* were administered with tBHP (0.5, 1 and 5 mM) followed by determination of brood size. The concentration range of tBHP was based on previous reports [5,10,12]. Compared to an un-administered control, 0.5 and 1 mM tBHP did not affect brood size in mock *C. elegans*, while 5 mM tBHP reduced brood size that was similar to *gspd-1*-knockdown *C. elegans* (Figure 2). This indicated that short-term administration of 5 mM tBHP is detrimental to embryos. Consistent with a previous result [5], *gspd-1*-knockdown *C. elegans* with elevated MDA exhibited a brood size reduction (Figure 2). These results suggest that lipid peroxidation can directly contribute to embryonic lethality.

### 2.3. tBHP Stimulated Germ Cell Apoptosis in C. elegans

**Figure 3:**
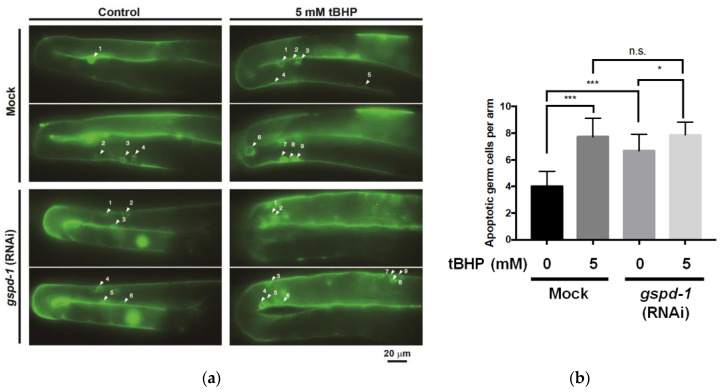
Short-term of tBHP administration on young adults of mock and *gspd-1*- knockdown *C. elegans* promoted germ cell apoptosis. (**a**) Fluorescent micrographs of apoptotic germ cells (white arrowheads) in the gonad of mock and *gspd-1*-knockdown *C. elegans* with or without 5 mM tBHP (scale bar 20 μm). (**b**) Mean apoptotic germ cells. Data from three independent experiments are shown as the mean ± SD (*n* = 3, n.s., not significant; * *p* < 0.05; *** *p* < 0.001).

To understand the tBHP effect in the germ cell, the GFP reporter strain *bcls39* was used to determine germ cell apoptosis by labeling the somatic sheath cell surrounding the apoptotic germ cells [13]. As shown in Figure 3a,b, mock *C. elegans* displayed a basal level of germ cell death (4.0 ± 1.1 apoptotic cells per gonad), whereas germ cell death of *gspd-1*-knockdown *C. elegans* was increased (6.7 ± 1.2 apoptotic cells per gonad). The lipid peroxidation induction using 5 mM tBHP enhanced germ cell death in mock *C. elegans* (7.7 ± 1.3 apoptotic cells per gonad). Moreover, 5 mM tBHP administration toward *gspd-1*-knockdown *C. elegans* promoted more apoptotic germ cells than without 5 mM tBHP administration (7.9 ± 0.9 apoptotic cells per gonad) (Figure 3b). These findings show that tBHP administration toward *C. elegans* stimulates germ cell apoptosis.

### 2.4. tBHP Increased Lipid Peroxidation in C. elegans

**Figure 4:**
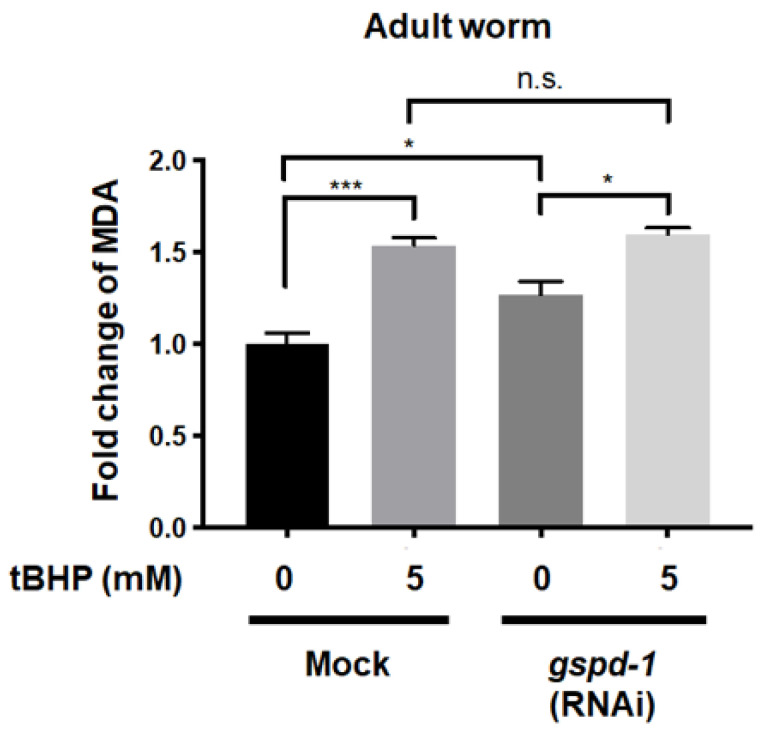
tBHP administration enhanced MDA levels in young adults of mock and *gspd-1*-knockdown *C. elegans*. The lipid peroxidation level of mock and *gspd-1*-knockdown *C. elegans* with or without 5 mM tBHP was determined. Data from three independent experiments are shown as the mean ± SD (*n* = 3, n.s., not significant; * *p* < 0.05; *** *p* < 0.001).

Lipid peroxidation derived from GSPD-1 deficiency has been associated with impaired embryogenesis [6]. Under the basal condition, the MDA level, a frequently measured biomarker of lipid peroxidation, was increased by 27% (*p* < 0.05) in *gspd-1*-knockdown *C. elegans* compared to mock *C. elegans* (Figure 4), while 5 mM tBHP increased the MDA level by 54% (*p* < 0.001) in administered *C. elegans* compared to the un-administered mock control. tBHP only increased the MDA level by 26% (*p* < 0.05) in *gspd-1*-knockdown *C. elegans* compared to the un-administered *gspd-1*-knockdown control (Figure 4). No significant difference in the MDA level after 5 mM tBHP administration was found between *gspd-1*-knockdown and mock *C. elegans*. These results indicate that tBHP induces lipid peroxidation in both mock and *gspd-1*-knockdown *C. elegans*.

### 2.5. tBHP Enhanced iPLA Activity in C. elegans

**Figure 5:**
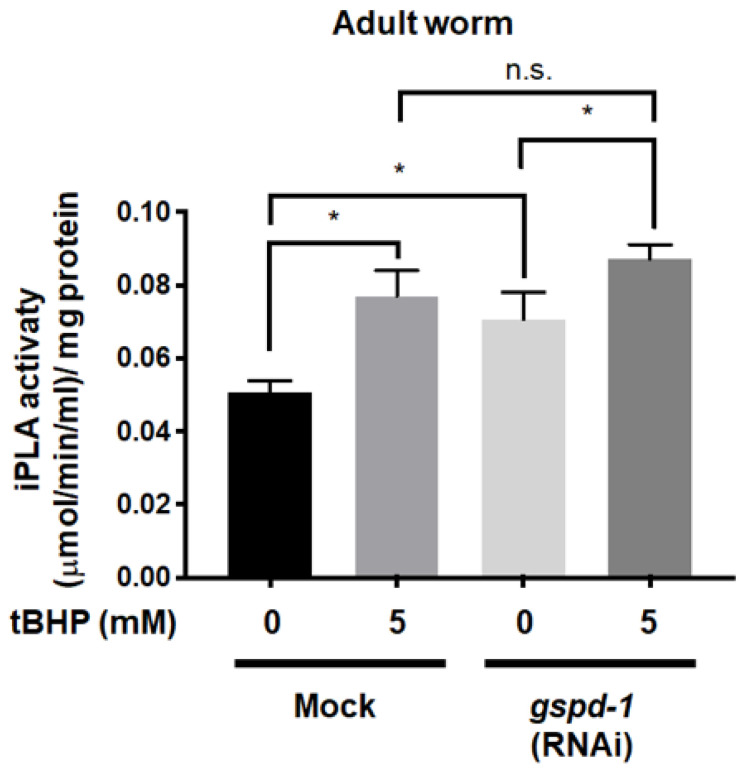
Increased iPLA activity in mock and *gspd-1*-knockdown *C. elegans* after short-term tBHP administration. The iPLA activity of mock and *gspd-1*-knockdown *C. elegans* with or without 5 mM tBHP was measured. Data from three independent experiments are shown as the mean ± SD (*n* = 3, n.s., not significant; * *p* < 0.05).

To further document that elevated lipid peroxidation could stimulate cytosolic calcium-independent phospholipase A_2_ (iPLA) [6], the iPLA activity was measured. Consistent with lipid peroxidation results, as shown in Figure 4, tBHP increased iPLA activity in mock *C. elegans* by 52% (*p* < 0.05) compared to the un-administered mock control, whereas tBHP promoted iPLA activity by 24% (*p* < 0.05) in *gspd-1*-knockdown *C. elegans* compared to the un-administered *gspd-1*-knockdown control (Figure 5). This figure also shows that *gspd-1*-knockdown *C. elegans* had enhanced iPLA activity by 40% (*p* < 0.05) compared to mock *C. elegans* and that no significant difference was observed in iPLA activity between *gspd-1*-knockdown and mock *C. elegans*, both of which were administered with 5 mM tBHP.

## 3. Discussion

**Figure 6:**
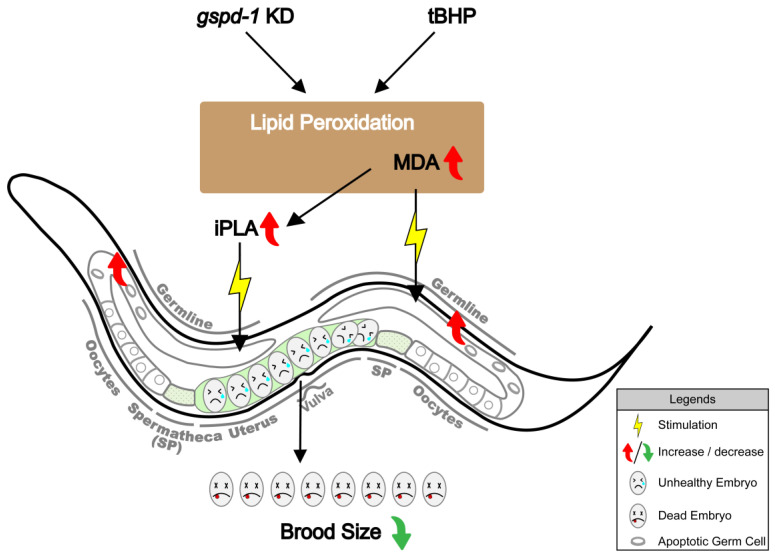
Proposed scheme of tBHP administration resembles GSPD-1 deficiency with increasing lipid peroxidation which can cause directly oxidative damage and enhances iPLA activity leading to increased germ cell death and eventual embryonic lethality.

The current study provides direct evidence to support the notion that lipid peroxidation promotes death of germ cells and impairs embryogenesis. All these abnormalities without exogenously added oxidant have been reported in GSPD-1 deficient *C. elegans* [5,6]. The altered phenotypes caused by short-term tBHP administration resemble GSPD-1 deficiency in *C. elegans* without the addition of exogenous peroxide [5]. Mechanistic studies indicate that disturbed oogenesis and embryogenesis in *C. elegans* could be attributed partly to tBHP induced lipid peroxidation as measured by MDA formation leading to stimulated iPLA activity and subsequent defective embryogenesis as proposed in Figure 6.

Elevated oxidative stress and oxidative damage have been considered as a main culprit for promoting death of germ cells and impaired embryogenesis in G6PD-deficient *C. elegans* [5,6]. An important function of G6PD lies in the regeneration of GSH by providing the reducing equivalent NADPH [14,15]. tBHP has been commonly used as a lipid hydroperoxide analog to evaluate the mechanism of biological alterations resulting from oxidative damage [10,11]. tBHP produces a peroxyl radical adduct in the absence of NADPH in rat liver microsomal fractions [16]. tBHP is metabolized to tert-butanol at the expense of glutathione, which can be depleted by oxidation to the disulfide form, GSSG [17]. tBHP rapidly depletes GSH in G6PD-deficient erythrocytes, while normal erythrocytes are unaffected [18]. Excessive lipid peroxidation induced by tBHP disrupts membrane and causes hemolysis [19]. GSH and ascorbate rapidly scavenge tBHP-derived radicals and protects membrane [20]. Supplementation of vitamins C and E partially restores GSH and reduces hemolysis in tBHP-administered erythrocytes [21]. These findings indicate that tBHP alters redox homeostasis, in part, by interfering with the GSH/GSSG balance.

Lipid peroxidation is increased in embryos of reduced GSPD-1 activity, which is linked to embryonic impairment [6]. However, a direct link between lipid peroxidation and embryonic lethality has not been clearly established in *C. elegans*. In the current study, tBHP induces lipid peroxidation as indicated by enhanced MDA production. Such increased lipid peroxidation is analogous to that found in GSPD-1-deficient embryos [6]. These findings provide a foundation for justifying the use of tBHP as an inducer of lipid peroxidation for investigating the downstream events of GSPD-1 deficiency and, subsequently, altered embryonic development.

Although tBHP elicits an oxidative stress response in *C. elegans* [22], how it causes embryonic defects via lipid peroxidation needs to be further delineated. tBHP has been proposed to diffuse into cytosol and forms radicals, which react with membrane lipids to initiate peroxidation [23]. The peroxidation of membrane phospholipids (polyunsaturated fatty acids) causes the formation of reactive aldehydes and membrane disruption, including disturbed membrane permeability and fluidity [24]. Such phenomena are in accordance with membrane-associated embryonic defects, including altered permeability, polarity and cytokinesis, as observed in GSPD-1-deficient *C. elegans* embryos [6]. These defects are linked to the disturbed membrane lipid composition caused by the activation of iPLA due to elevated lipid peroxidation in GSPD-1-deficient *C. elegans* embryos. tBHP and H_2_O_2_ have been reported to alter the composition of phospholipids in primary neocortical cells [25], which is analogous to the altered phospholipid profile in GSPD-1-deficient *C. elegans* adults [6].

In addition, phospholipase A_2_ (PLA_2_) has been reported to play a role in tBHP-induced cell death of other cells [26]. tBHP can induce PLA_2_-mediated hepatocyte cell death. An inhibitor of PLA_2_, mepacrine, blocks the release of arachidonic acid by tBHP, suggesting that activation of PLA_2_ is required for tBHP-induced cell injury [27]. The proposed involvement of iPLA is supported by the finding that tBHP or H_2_O_2_ induced neural cell death is mediated by iPLA activity [26]. An inverse correlation has been shown between iPLA activity and G6PD activity in *C. elegans* during embryogenesis [6]. In fact, oxidative damage, such as lipid peroxidation, has long been recognized to induce PLA_2_ activity, leading to altered lipid composition in membrane [28].

The proposed iPLA activation by tBHP in *C. elegans* is further supported by the findings that 5 mM tBHP increased iPLA activity in mock *C. elegans*, while this effect was less in GSPD-1 deficiency (Figure 5). Such findings are consistent with the postulate that, once the buildup of lipid peroxidation in germ cell death (Figure 3) and lipid peroxidation as measured by MDA formation (Figure 4) has reached a critical concentration, additional oxidative stress will have minimal effects in these situations. Although lipid peroxidation may be associated with impaired oogenesis [29], the link between lipid peroxidation and germ cell death has not been established in *C. elegans*. The increased MDA in *gspd-1*-knockdown *C. elegans* is consistent with a previous report that G6PD deficiency enhances oxidative stress as indicated by the ROS-sensitive dye DCFDA [5]. The cytotoxicity of tBHP has also been linked to oxidative stress and apoptosis in a similar manner as with severe G6PD deficiency [30,31,32,33,34,35,36,37]. Hence, tBHP enhanced MDA levels and stimulated iPLA activity by MDA in GSPD-1 deficient *C. elegans* are strong evidence indicative of a direct involvement of lipid peroxidation in germ cells death and impaired embryogenesis during embryonic development in *C. elegans*.

## 4. Materials and Methods

### 4.1. Worm Culture

N2 (wild type) *C. elegans* was acquired from *Caenorhabditis* Genetics Center (University of Minnesota, Minneapolis, MN, USA). A reporter strain of *bcIs39* [P*_lim-7_ced-1::gfp;lin-15*(+)] for detecting germ cell apoptosis [13] was a gift from Prof. Szecheng John Lo (Department of Biomedical Sciences, Chang Gung University, Taoyuan, Taiwan). Nematode strains were cultured on a nematode growth medium (NGM) agar plate seeded with bacterial lawn at 20 °C according to standard procedures [38].

### 4.2. gspd-1 RNAi Knockdown

RNAi knockdown was performed by feeding dsRNA-expressed bacteria according to a standard procedure [39]. In brief, gravid hermaphrodites fed on *E. coli* OP50 were administered with hypochlorite bleach. Eggs were incubated in M9 buffer overnight to obtain synchronized L1 larvae, which were cultured on NGM agar containing 1 mM IPTG, antibiotics (ampicillin and carbenicillin) (Sigma-Aldrich, St. Louis, MO, USA), and seeded with HT115 *E. coli* expressing L4440 vector control (mock) or a *gspd-1* RNAi described previously [5].

### 4.3. Reverse Transcription and Quantitative PCR (qPCR)

Total RNA of *C. elegans* samples were extracted using TRIzol^®^ (Invitrogen, Carlsbad, CA, USA). cDNA was synthesized from 1 μg of the total RNA by the use of an iScript cDNA synthesis kit (Bio-Rad, Hercules, CA, USA). Quantitative PCR was performed by using a Bio-Rad iQ5 and a SYBR Green Supermix reagent (Yeastern Biotech, New Taipei City, Taiwan). Primers for amplify *gspd-1* were as follows: forward primer, 5′-atgctcttgctgttgttcacatc-3′; reverse primer, 5′-cgctttaattcaccagacggatag-3′. The thermal cycle program was as follows: 95 °C for 10 min, 40 cycles of 95 °C for 15 s and 60 °C for 1 min. The expression level of *gspd-1* was normalized to threshold cycle (Ct) values of the housekeeping gene (beta-actin: forward primer, 5′-tcggtatgggacagaaggac-3′; reverse primer, 5′-catcccagttggtgacgata-3′).

### 4.4. tBHP Administration

For short-term tBHP administration, the L4 stage of mock or the *gspd-1*-knockdown *C. elegans* was collected by washing NGM plate with M9 buffer. The *C. elegans* samples were transferred to a 15 mL tube; soaked in 10 mL of M9 buffer containing 0.5, 1, or 5 mM tBHP (Sigma-Aldrich, St. Louis, MO, USA); and placed on a shaker for 30 min at room temperature. Subsequently, tBHP-administered worms were washed twice with the M9 buffer and centrifuged at 1500× *g* for 2 min to remove residual tBHP followed by transferring to a fresh NGM plate and kept overnight at 20 °C.

### 4.5. Brood Size Determination

Staged L4 *C. elegans* hermaphrodites were administered with tBHP and recovered on RNAi plates. These worms were transferred daily to fresh RNAi NGM plates during the egg-laying period. After 2–3 days, the viable progenies hatched from the eggs were counted by using a dissecting microscope (Nikon SMZ645, Tokyo, Japan). At least 20 worms were scored in each experiment.

### 4.6. Germline Apoptosis Assay

A reporter strain, *bcIs39* [P*_lim-7_ced-1::gfp*;*lin-15*(+)], was used to determine germ cell apoptosis by visualizing the signal of CED-1::GFP, which indicates the dying germ cell engulfed by the somatic sheath cell [13]. The number of apoptotic germ cells was quantified by counting the surrounding GFP signal in the gonad arm. Young adults of mock and *gspd-1*-knockdown *C. elegans* administered with the indicated concentrations of tBHP were anesthetized with 2% levamisole followed by mounting on a slide with a 2% agarose pad [5]. Fluorescent microscopic images were taken by using a fluorescence microscope (Leica DM 2500; Leica, Wetzlar, Germany) coupled with a CCD camera (Photometrics, Coolsnap K4, Tucson, AZ, USA) followed by analysis with imaging software (Image J 1.51j8 (Wayne Rasband, Bethesda, MD, USA)).

### 4.7. MDA Assay

Malondialdehyde was measured by using the Oxiselect thiobarbituric acid reactive substances assay kit (Cell Biolabs, San Diego, CA, USA). The *C. elegans* lysate was prepared according to a previous protocol [6]. A 50 μL worm pellet was used for a single test. Upon homogenization by sonication, a 100× BHT solution was added immediately to achieve a final concentration of 1× to prevent further oxidation of MDA in the sample. The lysate was then centrifuged at 10,000× *g* for 5 min. The supernatant was collected for the MDA assay according to the manufacturer’s protocol.

### 4.8. iPLA Assay

The iPLA activity was measured by using the cPLA_2_ assay kit (Cayman Chemical, Ann Arbor, MI, USA) with modification [6]. For determining iPLA activity, the assay buffer was substituted with a calcium-free buffer which consisted of 300 mM NaCl, 60% glycerol, 10 mM HEPES, 8 mM Triton X-100, 4 mM EGTA and 2 mg/mL of BSA. Synchronized *C. elegans* adults were washed from the NGM plate. After homogenization by sonication, worm lysates were centrifuged at 10,000× *g* for 15 min at 4 °C. The supernatants were collected for subsequent procedures according to a previous report [6].

### 4.9. Statistical Analysis

All statistical analyses were conducted using Prism 8.4.3 (471) version for MacOS (GraphPad 8, San Diego, CA, USA). Data of three independent experiments are presented as mean ± SD. The statistical difference between the control and experimental groups was analyzed by independent student’s *t*-test. *p*-values below 0.05 were considered statistically significant.

## Abbreviations

G6PD	glucose-6-phosphate dehydrogenase
NADPH	nicotinamide adenine dinucleotide phosphate
tBHP	*tert*-butyl hydroperoxide
MDA	malondialdehyde
iPLA	calcium-independent phospholipase A_2_
